# Biogas technology in fuelwood saving and carbon emission reduction in southern Ethiopia

**DOI:** 10.1016/j.heliyon.2020.e04791

**Published:** 2020-10-16

**Authors:** Getnet Alemu Desta, Yoseph Melka, Getachew Sime, Fikadu Yirga, Mequannt Marie, Mebrahtu Haile

**Affiliations:** a Natural Resource Management Program, Oda Bultum University, Ethiopia; b Natural Resources Economics and Policy Program, Wondo Genet College of Forestry and Natural Resources, Hawassa University, Ethiopia; c Department of Biology, Hawassa University, Ethiopia; d College of Dryland Agriculture and Natural Resources, Mekelle University, Mekelle, Ethiopia

**Keywords:** Adopter, Biogas energy, Deforestation, Fuelwood, Greenhouse gas emission

## Abstract

Most rural communities in developing countries, rely heavily on traditional biomass for cooking and lighting. Furthermore, a large area of forest land has been changed to other land-use types like agricultural land is becoming a serious problem in Wondo Genet district. This situation largely contributed to deforestation and forest degradation. Hence, assessing the efficiency of adopting an alternative source of energy was found to be very important. This study was carried out to examine the role of biogas technology in fuelwood saving and carbon emission reduction in Wondo Genet district, southern Ethiopia. The multi-stage sampling procedure was followed to select sample households. A total of 152 households (54 adopters and 98 non-adopters) were involved in the household survey. Moreover, 25 test subjects were taken randomly from both adoption categories to conduct Kitchen Performance Test. Descriptive statistics and independent-sample t-test were used to analyze the data. Results showed that the major fuel sources for domestic use were plantation forest, natural forest, crop residue, and animal dung, accounting 46.71 %, 30.92 %, 15.13 %, and 7.24 %, respectively. Among the 54 sampled biogas plants, 32 (59.26 %) were a digester size of 6 m^3^ whereas the remaining 22 (40.74 %) were of 8 m^3^. The annual fuelwood saving potential of the technology was found to be 1423.06 kg with an emission reduction potential of 2.1 tons of CO_2_ e per biogas plant annually. Accordingly, all functional biogas plants were estimated to reduce about 91.63 tons of carbon emission annually. Generally, the biogas was found to be a promising technology in combating the pressure on forest resources and mitigating climate change. Therefore, the energy sector of the country should encourage households to adopt biogas plants that have more than 8 m^3^ digester size to improve the fuelwood and carbon emission reduction potential.

## 1 Introduction

Energy plays an indispensable role in changing the lives of human beings. The global energy demand is increasing rapidly due to population growth and economic development, and about 88 % of this demand relies upon fossil fuels (Weiland, 2010). In 2018, an estimated 55.3 GtCO_2_e of greenhouse gases (GHGs) were emitted into the atmosphere annually where G20 members account for 78 percent (UNEP, 2019). The majority of G20 countries are developed countries. However, the impact of climate change is significant in poor peoples in developing countries whose adaptive capacity is very low (Field and Barros, 2014). Besides, deforestation and degradation of forests have a huge contribution to GHGs in many developing countries, particularly in sub-Saharan Africa (Hosonuma et al., 2012). They are considered as a major factor to aggravate climate change and health risks (Altizer et al., 2013; Johnson et al., 2013). Clean and renewable energy resources are being used as the major contributors to the global energy demand accounted for about 18.1% of total final energy consumption (GSR, 2019). In realizing sustainable development and climate-resilient green economy, Ethiopia has targeted clean and renewable energy like biogas energy. Ethiopia's government has initiated the Climate-Resilient Green Economy (CRGE) policy to combat climate change and to build a green economy that will help for realizing its ambition of reaching middle-income country before 2025. Under a conventional development path, greenhouse gas (GHG) emissions would be more than double from 150 Mt CO_2_e in 2010 to 400 Mt CO_2_e in 2030. However, Ethiopia's Climate-Resilient Green Economy (CRGE) initiative follows a sectoral approach which could help the country achieve its development goals by limiting GHG emissions to 150 Mt CO_2_e to 250 Mt CO_2_e (Ethiopian EPA, 2011).

Biogas is a renewable energy technology that utilizes human wastes, animal wastes, as well as municipal landfill wastes to produce a flammable methane gas which is important for cooking and lighting purposes (Arthur et al., 2011; Lansing et al., 2008). It is also clean and renewable energy which consists of methane (CH_4_) 60%–70% and carbon dioxide (CO_2_) 30%–40%, 1–5% hydrogen and traces of nitrogen, hydrogen sulphide, oxygen, water vapor, and slurry (Erdogdu, 2008). Biogas is produced by methanogenic bacteria acting on bio-digestible materials in the absence of oxygen in the process of anaerobic digestion (Harris, 2005). It contributes to minimizing the environmental impacts of GHG emissions, which results in climate change (Aggarangsi et al., 2013). The use of anaerobic digestion to create biogas from dairy manure and other organic wastes can reduce GHG emissions in two distinct ways. First, it stores and digests manure under anaerobic conditions and prevents the release of a greenhouse gas like methane (CH_4_) directly into the atmosphere. Second, the biogas generated by the anaerobic digestion process can replace the use of fuels that generate GHGs (Cuéllar and Webber, 2008).

Globally, fuelwood represents over 50 % of the total wood production from forests. Out of this, one-third of fuelwood is harvested unsustainably and leads to deforestation and forest degradation (Bailis et al., 2015). Besides, sub-Saharan Africa countries rely on fuelwood for 90–98% of the energy consumption (Idiata et al., 2013). Specifically, Ethiopia's energy consumption depends significantly on traditional biomass that accounts for about 91 % of the total energy consumption for cooking and lighting (MoWIE, 2012). In Wondo Genet district, most rural communities also rely extremely on traditional biomass energy. Furthermore, a large area of forestland has been changed to other land-use types due to overharvesting of the forests. This situation has caused a scarcity of fuelwood sources and forced to search for other options. The overall objective of this study was to investigate the contribution of biogas technology in fuelwood saving and carbon emission reduction in Wondo Genet district, southern Ethiopia.

## 2 Materials and methods

### 2.1 The study areas

**Figure 1:**
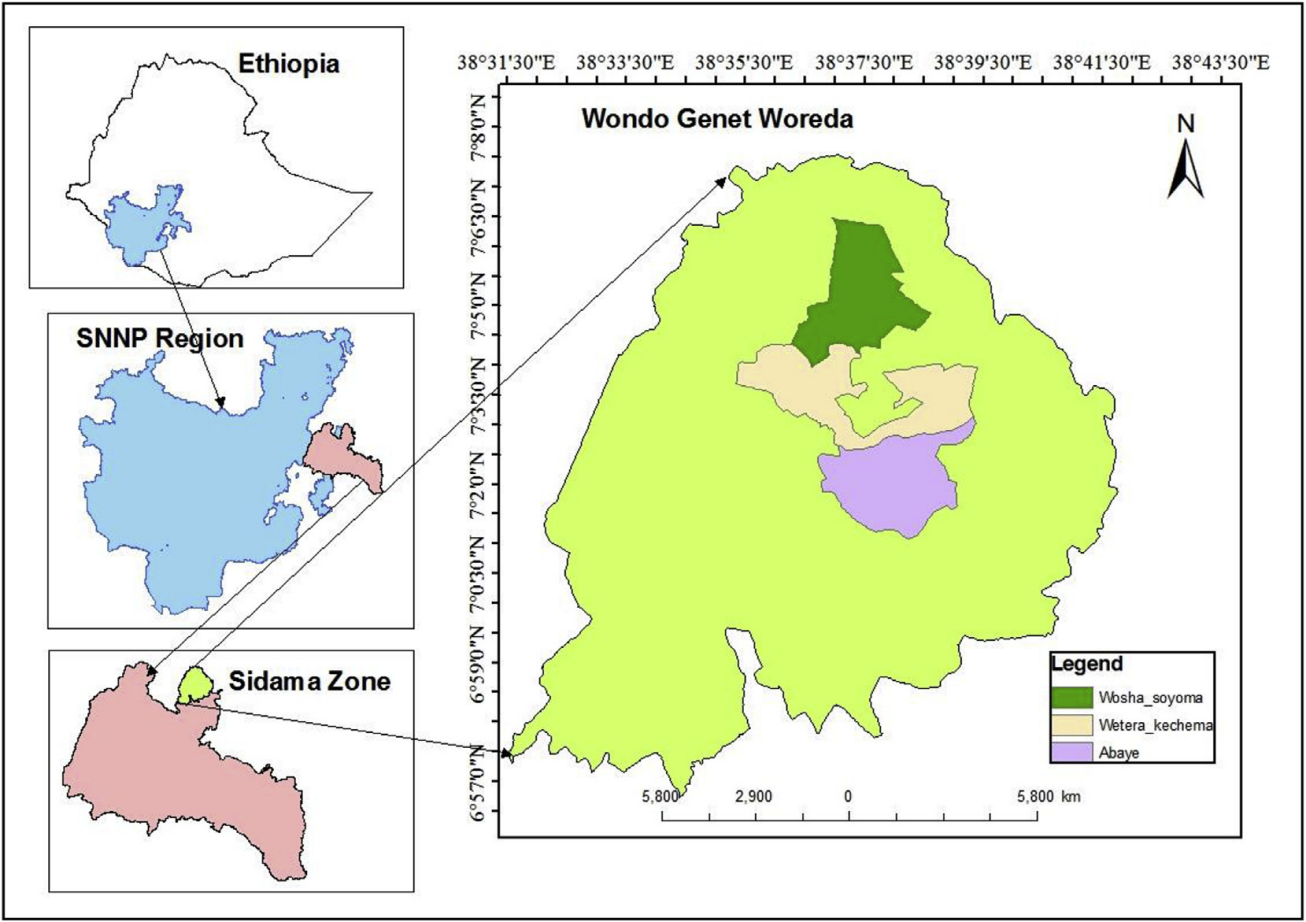
Map of the study area.

The study site, Wondo Genet district is located in the southeastern central highlands of Ethiopia, about 263 km far from Addis Ababa, at 6^o^57′0″N to 7^o^8′0″N latitude and 38^o^31′30″E to 38^o^43′30″E longitude (Figure 1). The elevation of the study site ranges between 1,600 and 2,580 m.a.s.l.

The population projection in 2016 showed that the total number of households and population size in Wondo Genet district to be 40,310 and 201,552, respectively. Out of this, 102,716 were male while 98,836 were females. Besides, the numbers of urban and rural residents were 46,584 and 154,968, respectively. Its total household number was estimated to be 40,310 (CSA, 2013). The district is rich in water resources and has several streams. Some of them include Wosha, Worqa, Hallo, and Lango. The vegetation is composed of partially disturbed natural forests, highly disturbed natural forests, and plantation forests (Ango, 2005). Moreover, the livelihood of the households is mostly dependent on smallholder agricultural farming with an average landholding size of less than one hectare per household. The major crops grown in the district includes *Enset* (*Enset ventricosum*), *khat* (*Catha edulis*), sugarcane (*Saccharum officinarum*), maize (*Zea mays*) and potatoes (*Solanum tuberosum*) (Dessie and Kleman, 2007).

### 2.2 Data sources

Both primary and secondary data sources were used. The primary data were collected from sample household survey, focus group discussion, key informants’ interview, and field observation. A complementary secondary data was collected from Water, Mineral and Energy office of the district, kebele administration offices, other published books and articles and unpublished theses and reports.

### 2.3 Sampling techniques and sample size

The multi-stage sampling procedure was followed to select adopters and non-adopter households. At the first stage, out of 16 kebeles found in Wondo Genet district, three kebeles were selected purposively with the level of adoption of biogas technology and proximity to the forest (less than 2 km). Thus, kebeles with higher domestication level and closer proximity to forests were selected. The first three kebeles having a higher number of biogas technology adoption were taken for this study. At the second stage, a list of biogas technology adopters and non-adopter household heads in the selected kebeles were obtained from the district and *Kebele* (the smallest administrative division in Ethiopia) administration offices. Then, the total sample size was determined for adopters and non-adopters of the technology separately. Accordingly, the number of sample households for both adopter and non-adopter of the target population were determined using the formula indicated in Israel (1992) at 92 % confidence level and 0.08 (8 %) level of precision;

(1) $$\text{n}=\frac{\text{N}}{1+\text{N}{\left(\text{e}\right)}^{2}}$$

Where; *n* is the sample size, *N* is the population size, and *e* is the level of precision at 92 % significance level.

In the third stage, the number of sample households from each selected *kebeles* were determined based on the Probability Proportional to Size (PPS) sampling technique (Table 1). Finally, a simple random sampling technique was used to select sample households from the three *kebeles*.

**Table 1:** Distribution of sample size in each selected kebele.

Kebeles	Household size		Sample size taken	
	Adopters	Non-adopters	Adopters	Non-adopters
Wotera-Kechema	21	2239	10	44
Abaye	42	1408	19	29
Wesha-Soyama	56	1194	25	25
Total	119	4841	54	98

### 2.4 Kitchen performance test procedure

The quantity of wood biomass that can be saved by using biogas technology was estimated based on the kitchen performance test (KPT). The KPT is a specific type of performance test which is used to measure fuel saved when cooks are changed from inefficient to efficient stoves (Bailis et al., 2007; Smith et al., 2007). Out of the 56 adopters of biogas technology in Wesha-Soyama kebele, 25 test subjects were selected using a random selection method. An equal amount of test subjects was also selected randomly from non-adopters of biogas technology for a cross-sectional study. As a rule of thumb, in a very small target population, mostly less than 200 families the number of families who can be involved in the initial survey should not less than 20 (Bailis et al., 2007). The sample size should be as high as possible during the high variation in the amount of fuel used and saved, which is often the case in KPTs. In this case, the starting point is the assumption of a typical variation, expressed as the Coefficient of Variation (CV).

Tests were properly conducted to get a reliable and genuine result. During KPTs, separate phases in isolated kitchens, having practically comparable socio-economic and cultural conditions were taken. According to Bailis et al., 2007, at least 3 days consecutive testing period is required. For undertaking KPT, the mass of wood for each sample household was pre-weighed at the beginning of the day and the remaining wood was weighed at the end of the day. Festivals or holidays were not considered since more wood is needed for cooking. Test subjects were informed to cook normally during the testing period. The aim was to capture their usual behavior in the kitchen. They were also informed to use fuel only from the stock that had been pre-weighed and they were visited at least once a day to check whether they are using only fuel from the weighted stock. Finally, a statistical analysis of the mean fuel savings estimation was conducted on the test results. Then, the precision for a sample of size *n* is determined using the formula as follow;

(2) $$\text{Precision }=1.67\times \;\frac{\text{SEy}}{\bar{\text{y}}}\;\times 100$$

where; $\bar{\text{y}}$ is the estimate of mean fuel savings, *SEy* is the standard error of the estimate, 1.67 is used as an approximation to the critical value t_0.95_, -1, which will vary between 1.75 and 1.64 as the sample size *n* increases from 15 to very large. In this KPT, CV for daily fuelwood consumption of biogas technology adopters and non-adopters were 0.23 and 0.13, respectively. Moreover, the precision attained was 24.8 %. This indicates that the sample size satisfies the 90/30 rule. When the sample sizes are large enough to satisfy the 90/30 rule, endpoints of the 90% confidence interval lie within +/- 30% of the estimated mean. Therefore, no additional sample size was required for KPT (Bailis et al., 2007).

Estimation of minimum sample sizes in simple random sampling was conducted using the following formula. Since the project (adopter) and baseline (non-adopter) samples are independent, then the standard error of the estimate is:

(3) $${\text{SE}}_{\text{y}}=\sqrt{\frac{{\text{s}}_{b^{2}}}{{\text{n}}_{\text{b}}}+\frac{{\text{s}}_{{\text{p}}^{2}}}{{\text{n}}_{\text{p}}}}$$

Where; s_b_ is the standard deviation of the i^th^ sample of baseline (non-adopters); s_p_ is the standard deviation of the i^th^ sample of the project (adopters); n_b_ is the sample size for non-adopters and n_p_ is the sample size of adopters. According to Bailis et al., 2007, the minimum required sample size to accomplish “90/*x*” precision with two independent samples is approximately equal to

(4) n˜≥(sb2+sp2y¯b−y¯p×1.67x100)2

n˜≥(0.562+0.2821.74−0.99×1.6730100)2n˜≥21.6

Hence, the total required sample size, in this case, is 44 test subjects. However, to reduce bias and make the samples more representatives, all the tested subjects (25 households from each group) were considered for this study.

Fuelwood use and saving were calculated in terms of kilograms per person per day. These were determined by dividing the kilogram per household per day by household size. The number of persons served on meals cooked during each day of the KPT was recorded through daily KPT survey. Moreover, weighting factors were used to calculate Standard Adult Equivalents (SAEs). The SAEs were determined using the guidelines for wood fuel surveys for the Food and Agricultural Organization (FAO) by Keith Openshaw cited in (Bailis et al., 2007) (Table 2). Finally, fuelwood consumption and saving were determined by per capita SAE (see Table 3).

**Table 2:** Standard adult equivalence factors.

Gender and Age	Fraction of standard adult
Child: 0–14 years	0.5
Female: over 14 years	0.8
Male: 15-59	1
Male: over 59 years	0.8

**Table 3:** Parameters for calculating carbon emission.

Parameter	Value	Source
Annual wood saving per biogas plant	KPT	Field survey
Emission factor of fuelwood	112 CO_2_ t/TJ	(Eggleston et al., 2006)
Net calorific value of fuelwood (wet basis)	15 MJ/kg	(Eggleston et al., 2006)
Conversion CO_2_/C	3.667	Ratio of molecular weights
Fraction of non-renewable fuelwood	88 %	(UNFCCC, 2012)

### 2.5 Estimation of carbon emission reduction from biogas utilization

The potential of biogas technology adoption in carbon emission reduction was assessed by estimating total fuelwood savings attained by biogas plants. As indicated by Houghton et al. (1997), it was calculated based on the net calorific values, emission factors of fuelwood and carbon storage in forests by using the formula;

(5) ERy=By,savings×fNRB,y×NCVbiomass×EFprojected−fossilfuel

Where:

ERy is emission reduction during the year in tons of carbon dioxide equivalent (tco_2_e).

B_y_, _savings_ is the quantity of woody biomass that is saved in tons or kilograms per biogas plant.

f_NRB,y_ is the fraction of woody biomass saved during the year that can be established as non-renewable biomass.

NCV_biomass_ is the net calorific value of the non-renewable biomass.

EF_projected-fossil fuel_ is an emission factor for the substitution of the non-renewable woody biomass by similar consumers.

Finally, the CO_2_e was converted to carbon using a conversion factor of 3.667 (ratio of molecular weights of CO_2_ and C).

## 3 Results and discussions

### 3.1 Socioeconomic and demographic characteristics of households

The results in Table 4 revealed that the average age of adopters and non-adopters were 47 and 43, respectively. The mean age difference was found to be statistically significant at 5 % significance level. The average family size and standard deviation of adopter sample households were 7.67 and 2.07 persons, respectively. On the other hand, the average family size and standard deviation for non-adopter households were 6.29 and 1.92, respectively. The t-test result showed that there is a significant difference between adopter and non-adopter sample households at 1 % significance level. The total farm size owned by households indicates the economic status of farmers. As a result, the household survey result showed that the average farm holding by adopter households was about 0.48 ha while it was 0.47ha for the non-adopter households. The mean difference between the two categories was found to be statistically insignificant. This may be related to the fact that both adopter and non-adopter sample households were taken from the area having almost similar socio-economic characteristics. Besides, the types of livestock owned by the sample households were converted into Tropical Livestock Unit (TLU) to compare livestock holding size between households. Accordingly, the independent sample t-test result showed that there is a significant mean difference in TLU between the two adoption categories at 1 % level of significance (Table 4).

**Table 4:** Distribution of respondents by household characteristics.

Variables	Adopters		Non-adopters		t-value
	Mean	SD	Mean	SD	
Age (year)	47	11	43	8	2.64∗∗
Family size (number)	7.67	2.07	6.29	1.92	4.12∗∗∗
Farm size (ha)	0.48	0.25	0.47	0.23	0.38
TLU	5.08	2.21	1.94	1.91	9.17∗∗∗

∗∗∗ and ∗∗ shows significant variation at1% and 5% level of significance.

The results in Table 5 revealed that the average annual incomes of the adopter and non-adopter households were 1271 and 1242.9 USD, respectively. The average family size was 6.56 and 7.08 persons for adopters and non-adopter households, respectively. Besides, standard adult equivalents of adopter and non-adopter sample households were found to be 4.92 and 5.04, respectively. In this regard, the results from the independent sample t-test on the two adoption categories were found to be statically insignificant (Table 5). This may be related to the fact that both adopter and non-adopter sample households were taken from the area having almost similar socio-economic characteristics.

**Table 5:** Average annual income, household size, and standard adult equivalents.

Variables	Adopters (n = 25)	Non-adopters (n = 25)	p-value
	Mean ± SD	Mean ± SD	
Annual income (USD)	1271 ± 575	1242.9 ± 527	0.86
Family size (number)	6.56 ± 2.02	7.08 ± 2.04	0.37
Standard adult equivalent	4.92 ± 1.5	5.04 ± 1.53	0.8

### 3.2 Fuel sources

**Figure 2:**
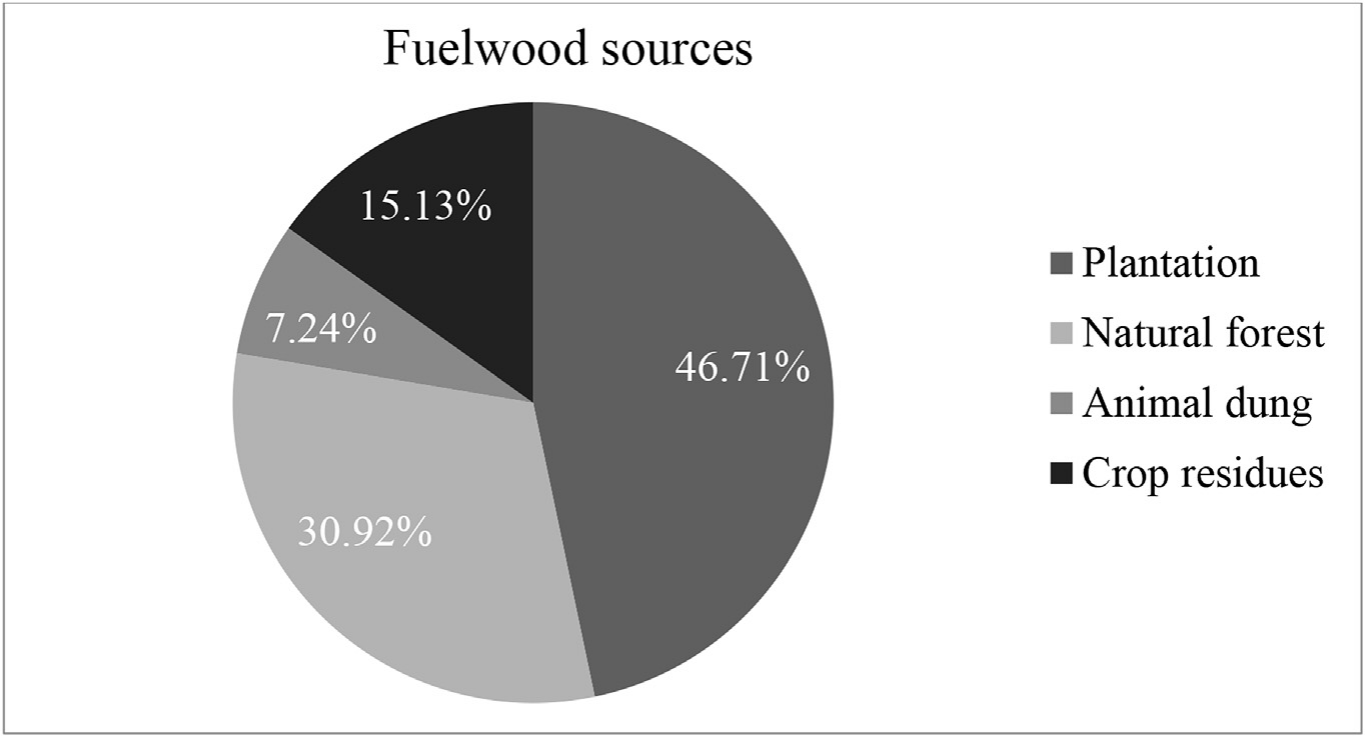
Distribution of households by their fuel source.

The results in Figure 2 revealed that from the total 152 respondents, about 71 (46.71 %) used plantations (homestead trees and public plantation forests) as a major source of fuel. Natural forest, which is collected illegally, was the second major fuel source for about 47 (30.92 %) of the households as their domestic energy consumption depends on it. Crop residues and cow dung were, however, the least commonly used fuel sources as used by 23 (15.13 %) and 11 (7.24 %) of households, respectively (Figure 2). A similar finding was reported in Wondo Genet catchment area that the proportion of plantations, natural forest as well as both crop residues and animal dung were 54 %, 19 %, and 17 %, respectively (Bekele et al., 2013).

### 3.3 Digester size and feedstock type of biogas plants

Among the 54 sampled biogas plants, 32 (59.26 %) were a digester size of 6 m^3^ whereas the remaining 22 (40.74 %) were of 8 m^3^ digester size. No biogas plant with 10 m^3^ or larger size was found in the studied sites (Table 6). This indicates that the dissemination of biogas plants, especially with larger digester sizes, is not satisfactory since Wondo Genet is endowed with huge water and livestock potential. About 79.63% of respondents used both cow-dung and latrine. The proportions of households used only cow dung; latrine and household wastes were 18.52%, 1.85%, and 0%, respectively. Interviews with respondents and focus group discussants have witnessed that the use of latrine together with animal dung is vital to maximize the energy produced from biogas technology as compared to using only animal dung. Latrine can supplement the shortage of cow dung. A previous study by (Lulie (2010)) in Ethiopia indicated that among 91 institutions visited, 59 (64.9%) have connected their digester to the toilets. On the other hand, 26 (28.6%) households have fed their digester with cattle dung alone, whereas 24 (26.4%) have combined cattle dung with human, kitchen, and other animal wastes. However, field observations and interviews witnessed that none of the biogas technology adopters in Wondo Genet area use kitchen wastes for biogas energy production.

**Table 6:** Digester size of adopted biogas plants.

Biogas digester's size	Frequency	Percent
6 m^3^	32	59.26
8 m^3^	22	40.74
10 m^3^ or larger	0	0
Total	54	100

### 3.4 Household daily fuelwood consumption

The results in Table 7 revealed that average daily per household fuelwood consumption was found to be 4.95 kg for the adopter and 8.34 kg for non-adopter tested subjects. Besides, the average adult mean equivalents served from cooked meals within 24 h were 5.09 for the adopter and 5.14 for non-adopter tested subjects. The average per capita fuelwood consumption of the adopter and non-adopter households were found to be 0.99 and 1.74 kg, respectively. The average daily and per capita fuelwood consumption of the adopters and non-adopters were found to be statistically significant (Table 7).

**Table 7:** Household daily and per capita fuelwood consumption (kg).

Variable	N	Aver. AME served	Aver. fuelwood used	Per capita fuelwood
		(HH/day)	(Kg/HH/day)	used (kg/AME/day)
Non-adopters	25	5.14 ± 1.49	8.34 ± 2.20	1.74
Adopters	25	5.09 ± 1.48	4.95 ± 1.67	0.99
t-value			6.11∗∗∗	5.96∗∗∗

∗∗∗ indicate significant variation at 1 % significance level along the column.

Moreover, it shows a 43.1 % fuelwood saving potential of the biogas technology as compared to the traditional three-stone fire stove. The annual per capita fuelwood consumption was 635.1 kg for non-adopter and 361.35 kg for adopter households. The annual fuelwood consumptions per household were 3207.25 kg for non-adopters and 1777.84 kg for adopters of biogas technology. Accordingly, the annual fuelwood savings from adopting biogas technology per household per year was found to be 1423.06 kg, which justifies the fuelwood reduction potential (44.37 %) of biogas technology adoption. The result of this study was supported by the finding in Kenya which shows that the average monthly fuelwood consumption by the adopter and non-adopter households were 187.5 and 228.5 kg, respectively (Wamuyu, 2014). As a result, about 1519.2 kg of fuelwood was saved per annum by using biogas, implying its role in the conservation of forest resources. It also agrees with the finding of Jetter et al. (2012) which revealed that cooking in rudimentary stoves and open fires has about 10–20 % conversion efficiency, prompting high primary energy consumption. Advanced wood burning and biogas stoves can potentially lessen biomass fuel utilization by 60 % or more (Jetter et al., 2012).

Furthermore, the result of the present study is less than the findings of (Amare, 2015) which indicated that households used 3,596.4 kg of fuelwood per annum before the installation of biogas plants and an average of 1062 kg of fuelwood per annum after installation of biogas plant which results in a reduction of 2,534.4 kg (equivalent to 70.47 % fuelwood consumption reduction per household year). The difference may be arising from temperature variation inside the digester, manure loading rate as well as from not considering fuelwood consumption in per capita SAE basis while estimating the amount of fuelwood saved by using biogas technology.

### 3.5 Role of biogas technology in carbon emission reduction

The quantitative fuel consumption survey showed that each biogas plant saves average fuelwood of 1423.06 kg (1.42 t) per year as compared to the traditional three-stone fire. The CO_2_ emission reduction potential of a biogas plant was estimated by using the net calorific value of fuelwood (wet basis) (15 MJ/kg), the emission factor of fuelwood (0.112 tCO_2_/TJ) and a fraction of non-renewable fuelwood (88 %). In this regard, about 2.1 t CO_2_ e was reduced per biogas plant per year. Therefore, from the 160 functional biogas plants in the study area, a total of 336 tons of CO_2_e emission was reduced per year. As a result, 91.63 tons of carbon can be saved per annum from these biogas plants in the area. However, if the adopters were using the biogas energy for baking bread and ‘*Injera’*, the carbon emission reduction is more than the current value.

The result of the present study is less than the finding of Mengistu et al. (2015) which reported a relatively higher amount of average GHG emission reduction per domestic biogas installation (about 5 tons of CO_2_e per year). The reason could be associated with the efficiency of the biogas plants under consideration in generating biogas energy. Moreover, nearly equal result with the present study is reported in the empirical study by Dresen et al. (2014) which showed that each improved cooking stove (ICS) in use leads to the average emission reduction potential of 2.145 tons of CO_2_ per ICS per annum. On the other hand, the result of the present study is higher than the finding of Liu et al. (2013) which reported that the average annual GHG emission saving potential of biogas technology was 1.3 tons. In the future, if 5 % of the total households (5 % of 40,310 households) could have access to install biogas plants in Wondo Genet district, about 4234 tons of CO_2_e or 1155 tons of carbon emission reduction can be attained per annum.

## 4 Conclusions

Most of the households are using fuelwood as a major source of energy for cooking in the study area. On the other hand, switching of the energy use to biogas makes adopters of the technology to consume less amount of fuelwood which results in reduced pressure on deforestation and forest degradation. Moreover, the demand for most households to adopt baking stoves for baking Injera was highly encouraging. The reason behind this is to reduce their reliance on fuelwood. The average fuelwood consumption per household per day and the average per capita fuelwood consumption per day showed a significant difference between adopters and non-adopters of biogas technology. The habit of using household wastes (including food wastes) not common in the study site. This would have a great impact on the amount of biogas produced. Furthermore, the result obtained from this study showed a fuelwood saving and carbon emission reduction potential of 1423.06 kg and 2.1 tons of CO_2_e per biogas plant per annum, respectively. This shows a substantial role of biogas technology in reducing carbon emission and the impact of climate change. Generally, the biogas plant was found to be a promising technology for rural energy security, forest conservation, and carbon emission reduction.

## 5 Policy implications

The governmental and non-governmental organizations working in the energy sector should try to address baking stoves for those households who have already adopted biogas technology. So that it would have a substantial impact in reducing the pressure on the natural and plantation forests degradation and maximizing the carbon emission reduction potential of biogas technology in the district. The biogas energy sector of the country should encourage households to adopt biogas plants that have more than 6 and 8 m^3^ digester size currently in an application so that the carbon emission reduction potential of the biogas technology would be improved. This would help to realize Ethiopia's Climate-Resilient Green Economy (CRGE) policies which focusses to reach its ambitious growth targets while keeping greenhouse gas emissions low. Policies in the energy sector should also focus on using food waste together with animal waste for increasing biogas production since the amount of biowaste is not sufficient for satisfying energy demand in the study site. Besides, the experts in the biogas energy sector should work in consortium with experts in agriculture, forestry, and environment on sustainable biogas technology adoption to reduce deforestation and mitigating climate change. Further investigations should focus on investigating the amount of methane emission reduced by using biogas technology. Since a significant amount of emissions are reduced from changing the manure to useful energy. Further study is also needed on the welfare effect of biogas technology adoption in the study area.

## Declarations

### Author contribution statement

Getnet Alemu Desta: Conceived and designed the experiments; Performed the experiments; Analyzed and interpreted the data; Wrote the paper.

Yoseph Melka, Getachew Sime, Fikadu Yirga, Mequannt Marie, Mebrahtu Haile: Analyzed and interpreted the data; Wrote the paper.

### Funding statement

This research did not receive any specific grant from funding agencies in the public, commercial, or not-for-profit sectors.

### Competing interest statement

The authors declare no conflict of interest.

### Additional information

No additional information is available for this paper.
